# Pre-capture multiplexing improves efficiency and cost-effectiveness of targeted genomic enrichment

**DOI:** 10.1186/1471-2164-13-618

**Published:** 2012-11-14

**Authors:** A Eliot Shearer, Michael S Hildebrand, Harini Ravi, Swati Joshi, Angelica C Guiffre, Barbara Novak, Scott Happe, Emily M LeProust, Richard JH Smith

**Affiliations:** 1 Department of Otolaryngology – Head & Neck Surgery, University of Iowa Carver College of Medicine, Iowa City, IA, 52242, USA; 2 Department of Molecular Physiology & Biophysics, University of Iowa Carver College of Medicine, Iowa City, IA, 52242, USA; 3 Agilent Technologies, Cedar Creek, TX, USA; 4 Agilent Technologies, Santa Clara, CA, USA; 5 Interdepartmental PhD Program in Genetics, University of Iowa Carver College of Medicine, Iowa City, IA, 52242, USA

**Keywords:** Massively parallel sequencing, Next-generation sequencing, Genomics, Targeted genomic enrichment, Sequence capture, Pre-capture multiplexing, Post-capture multiplexing, Indexing

## Abstract

**Background:**

Targeted genomic enrichment (TGE) is a widely used method for isolating and enriching specific genomic regions prior to massively parallel sequencing. To make effective use of sequencer output, barcoding and sample pooling (multiplexing) after TGE and prior to sequencing (post-capture multiplexing) has become routine. While previous reports have indicated that multiplexing prior to capture (pre-capture multiplexing) is feasible, no thorough examination of the effect of this method has been completed on a large number of samples. Here we compare standard post-capture TGE to two levels of pre-capture multiplexing: 12 or 16 samples per pool. We evaluated these methods using standard TGE metrics and determined the ability to identify several classes of genetic mutations in three sets of 96 samples, including 48 controls. Our overall goal was to maximize cost reduction and minimize experimental time while maintaining a high percentage of reads on target and a high depth of coverage at thresholds required for variant detection.

**Results:**

We adapted the standard post-capture TGE method for pre-capture TGE with several protocol modifications, including redesign of blocking oligonucleotides and optimization of enzymatic and amplification steps. Pre-capture multiplexing reduced costs for TGE by at least 38% and significantly reduced hands-on time during the TGE protocol. We found that pre-capture multiplexing reduced capture efficiency by 23 or 31% for pre-capture pools of 12 and 16, respectively. However efficiency losses at this step can be compensated by reducing the number of simultaneously sequenced samples. Pre-capture multiplexing and post-capture TGE performed similarly with respect to variant detection of positive control mutations. In addition, we detected no instances of sample switching due to aberrant barcode identification.

**Conclusions:**

Pre-capture multiplexing improves efficiency of TGE experiments with respect to hands-on time and reagent use compared to standard post-capture TGE. A decrease in capture efficiency is observed when using pre-capture multiplexing; however, it does not negatively impact variant detection and can be accommodated by the experimental design.

## Background

Massively parallel sequencing has expanded the genomics era by dramatically reducing the cost and time of large-scale DNA sequencing. Although whole genome sequencing may soon become routine, in terms of cost, time, and labor, it is often more practical to target specific regions of interest in the genome. Targeted genomic enrichment (TGE), also known as targeted sequence capture, allows efficient isolation of genomic regions prior to massively parallel sequencing [1]. Briefly, DNA libraries are hybridized with DNA or RNA oligonucleotides complementary to regions of interest (baits), and these bait-library complexes are pulled out of solution after the hybridization to generate an enriched library for sequencing. This method has generally been used to target exonic and splice-site sequences of the human genome, as ~ 85% of known mutations reside in these regions [2].

TGE has been used to discover mutations in sets of genes associated with specific diseases [3-5] or, in an unbiased way, by targeting the whole exome [2]. A trade-off exists between the number of base-pairs targeted for sequencing and the throughput of sequencing with respect to cost and time. However, in all types of TGE, there is increased efficiency in uncovering disease-causing mutations when compared to whole genome sequencing.

The increase in sequencer output has well outpaced our ability to efficiently use the sequence generated. While it is clear that saturating levels of sequencing coverage are required for the lowest false positive and false negative rates in TGE experiments [6], it is also clear that there is a diminishing return after the threshold coverage level for variant detection is exceeded, and, in fact, over-coverage can introduce errors [7]. Pooling samples is an attractive option to maximize sequencer output; however, due to sequencer error rate, reliable differentiation of true positives from false positives is generally difficult [8] unless specialized software is employed [9,10]. To avoid this difficulty, molecular barcodes (indexes) can be ligated to sheared DNA fragments prior to pooling in a process called multiplexing. Because the purpose of TGE is to focus on relatively small genomic regions of interest, multiplexing can be used to maximize sequencer output.

When applied to TGE, multiplexing can be performed prior to capture (pre-capture multiplexing) or after capture (post-capture multiplexing). The first protocols using molecular barcodes employed a post-capture approach [11] and post-capture multiplexing has since become the standard method for TGE. Several studies, however, have shown that the pre-capture approach is also feasible and that this method offers three important advantages over post-capture multiplexing: 1) decreased cost as the capture step is generally the most costly step in the TGE protocol; 2) reduced hands-on time as samples are pooled earlier in the protocol; and, 3) reduced cross-contamination risk by the earlier addition barcodes [12-15].

However, the limits of pre-capture multiplexing have not been thoroughly tested. Of two pilot studies showing the feasibility of pre-capture multiplexing one used 8 samples and single pools of 3 or 5 samples pooled pre-capture [12], while another used 9 samples with single pools of either 3 or 9 samples pooled pre-capture [13]. Both of these studies included only 1 pool at each pooling size and so a detailed comparison of the effects of increasing pool size and a comparison to standard post-capture pooling was not possible. In another study, pre-capture pools of 4, 6, and 12 were evaluated and the authors went on to use 6 pools of 8 samples to sequence 48 samples [14]. And finally, in another study the authors pooled 20 samples pre-capture, however, only a single pool was used and micro-array based TGE was used [15]. All of these studies showed the feasibility of pre-capture pooling for solution-phase TGE, however we sought to study the effects of pre-capture pooling on TGE in a systematic fashion in different pool sizes on a large number of samples.

In general, post-capture multiplexing is used for TGE whereas pre-capture multiplexing is not. In this study, we sought to address several questions germane to multiplexing during TGE experiments, including the effect of inter-sample competition for capture baits during hybridization, the impact on capture efficiency, and the downstream effects on overall sequence quality as measured by read mapping and duplicate reads. To address these questions, we compared standard post-capture multiplexing to pre-capture multiplexing using two pool sizes (n=12 or 16 samples per pool) to study a large set of samples (n = 96). We demonstrate the significant advantages of pre-capture multiplexing in cost and time reductions while at the same time maintaining our minimum threshold for accurate variant detection.

## Results

**Figure 1:**
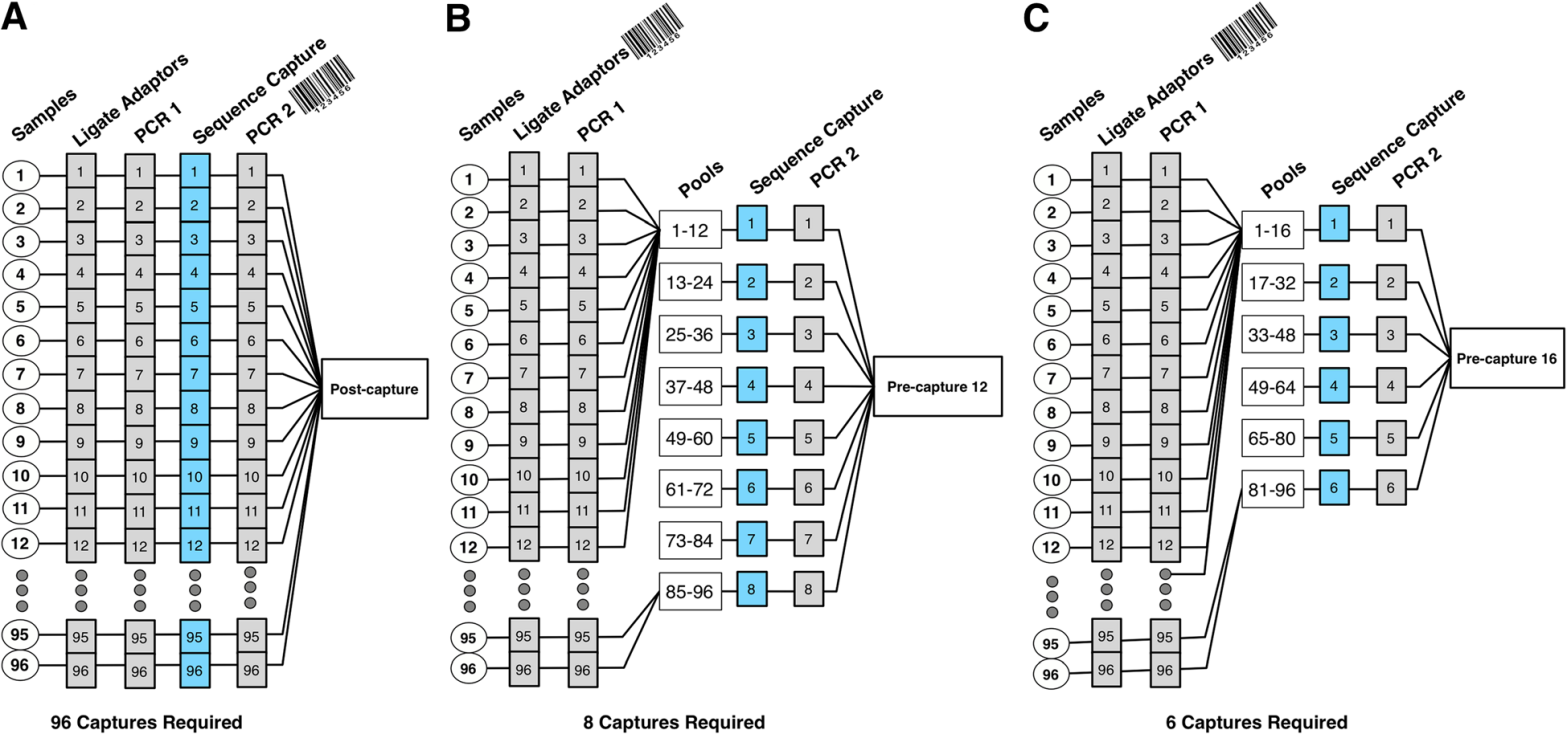
**Experimental design showing pertinent steps in the TGE protocol including the indexing PCR during which barcodes are incorporated into separate DNA libraries.A**) standard post-capture TGE, 96 captures are required as barcodes are incorporated during second PCR after capture and prior to sequencing, **B**) pre-capture multiplexing with pools of 12 (pre-capture 12a and pre-capture 12b), 8 captures required as barcodes are incorporated when adaptors are ligated prior to the first PCR and capture, and **C**) pre-capture multiplexing with pools of 16 (pre-capture 16), 6 captures required.

We compared standard post-capture multiplexing with pre-capture multiplexing for TGE of a relatively small genomic region of 521,647 bp that comprises all known non-syndromic deafness and Usher syndrome genes as described previously [3]. We performed TGE on a set of 96 DNA samples: 48 unknowns, 39 positive controls with 44 Sanger-sequence-verified deafness-causing mutations, and 9 negative controls. The samples were captured in two ways for comparison, as shown in Figure 1. (1) 96 samples were prepared using standard post-capture multiplexing TGE and run in a single Illumina HiSeq lane (post-capture 96) and (2) pools of 12 or 16 (pre-capture 12 and pre-capture 16) samples were pre-capture multiplexed with barcodes added during ligation and sequenced in groups of 96 in a single Illumina HiSeq lane (8 pools of 12 samples for pre-capture 12; 6 pools of 16 samples for pre-capture 16). TGE for post-capture samples was performed with the Agilent SureSelect XT version 1 protocol while TGE for pre-capture samples was performed with the Agilent SureSelect XT version 2, a protocol optimized for pre-capture pooling with regards to hybridization, amplification and capture wash conditions (see Methods). A single sample in the post-capture 96 set failed sequencing QC and was excluded from further analysis.

### Total reads and read mapping

**Figure 2:**
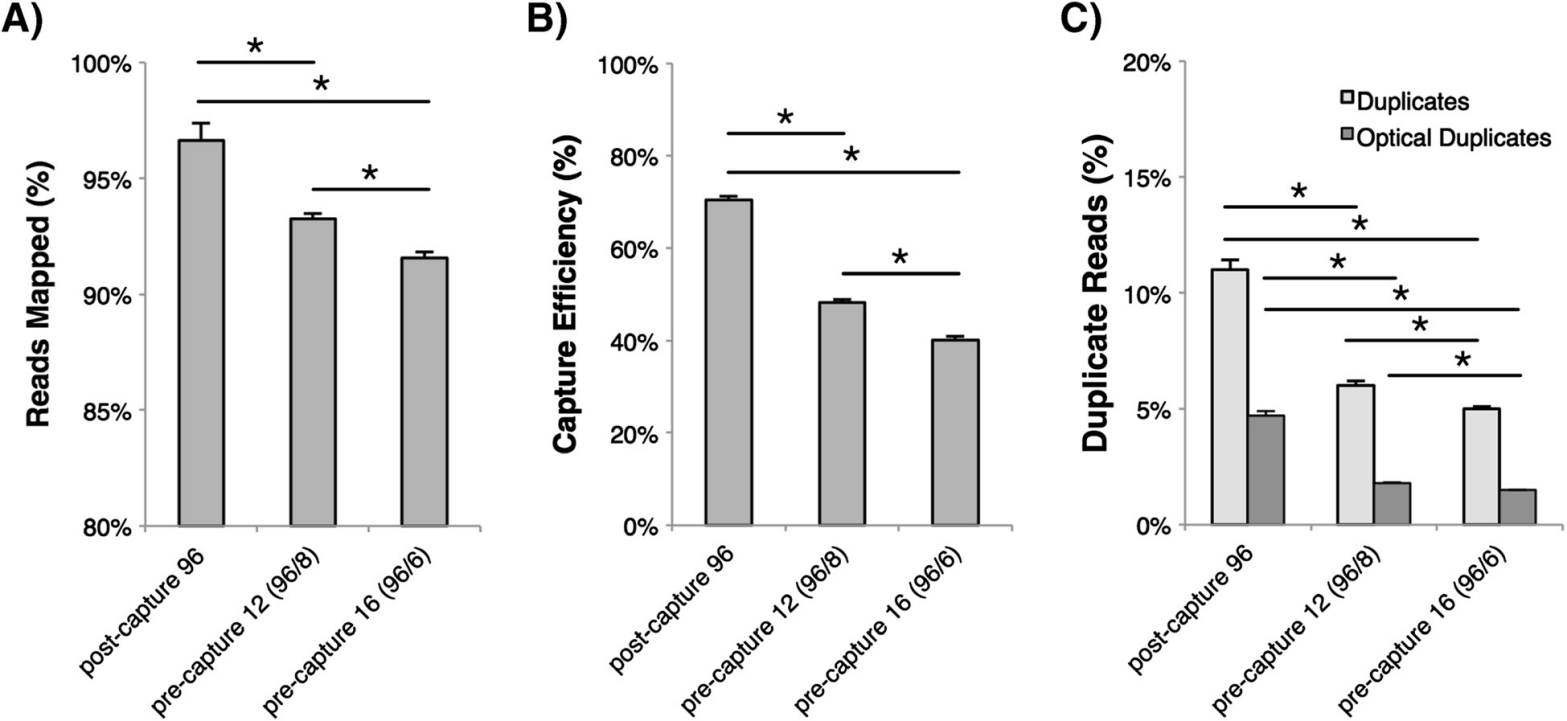
**Sequencing performance.** Average results for 96 samples run in a single lane using different multiplexing methods. Bars show standard error from the mean. **A**) Average percent of reads mapped to the reference human genome using BWA per multiplexing method, **B**) Average capture efficiency, as defined by the percentage of mapped sequencing reads overlapping targeted intervals, and **C**) Average percent of all reads identified as duplicates and optical duplicates by Picard tools. * p < 0.01 (independent samples *T*-test).

The total number of sequencing reads was significantly lower in pre-capture lanes as compared with the post-capture lane (Tukey’s post-hoc ANOVA p=0.004 and p=0.02 for pre-capture 12 and pre-capture 16, respectively), however, the two pre-capture lanes were not significantly different. 97.4%, 93.3%, and 91.6% of sequencing reads mapped on average for post-capture, pre-capture 12, and pre-capture 16, respectively (Figure 2A). The percent of mapped reads was significantly lower for pre-capture lanes (independent samples *T*-test p < 0.01 for both pre-capture 12 and 16), however in all cases, greater than 90% of reads mapped, constituting a high-quality sequencing run in our experience.

### Capture efficiency and duplicate reads

We define capture efficiency as the percent of all mapped reads that overlap the targeted regions. In our experience using SureSelect TGE, capture efficiency ranges from ~40% for small target regions (<200kb) to up to ~80% for large target regions (50 Mb, i.e. the exome) when using standard post-capture multiplexing (data not shown). On average, the capture efficiency for post-capture samples was 68.7% (Figure 2B). This was significantly higher (p < 0.01) than the average for both pre-capture 12 samples (45.3%) and pre-capture 16 samples (37.1%). The difference between pre-capture 12 and pre-capture 16 was also significantly different (p < 0.01). The difference in average duplicate reads and average optical duplicate reads also varied significantly between all three methods (p < 0.01 in all cases) as shown in Figure 2C, and was 12.6% versus 4.7% (post-capture), 7.1% versus 1.8% (pre-capture 12), and 5.8% versus 1.5% (pre-capture 16), respectively.

### Coverage performance

**Figure 3:**
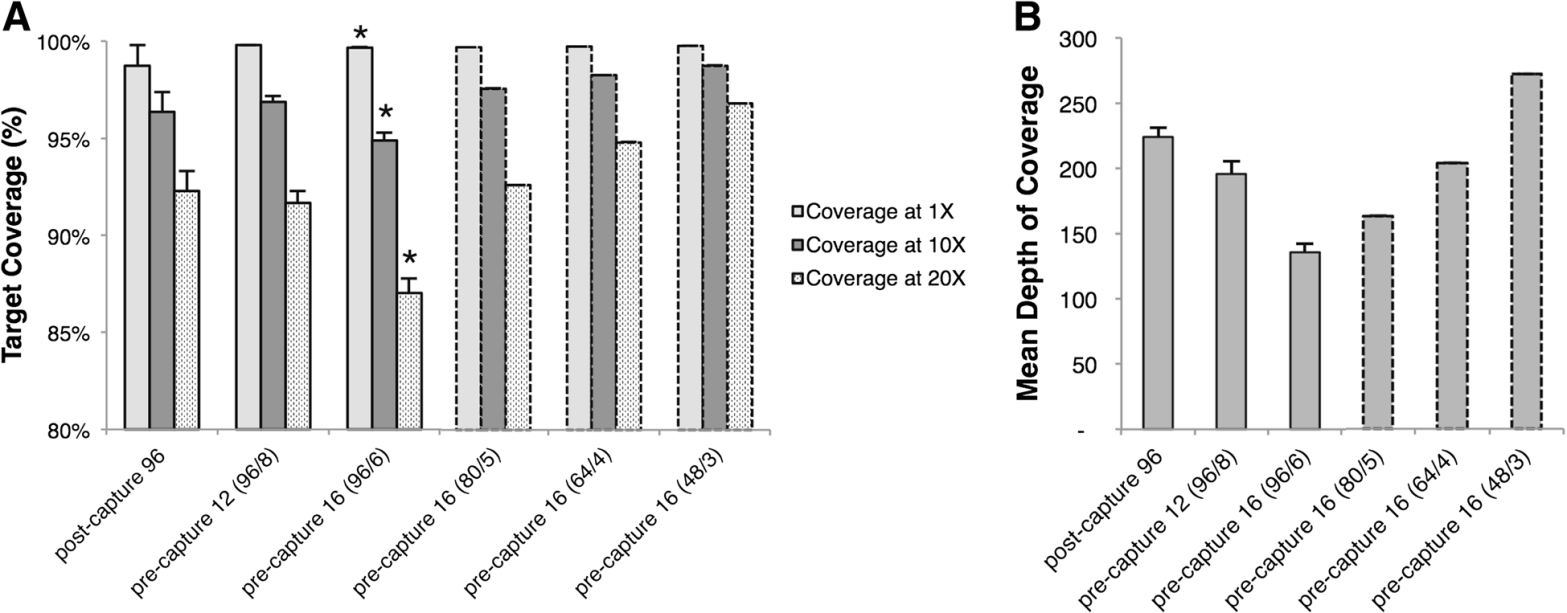
**Targeted region coverage statistics.** Average results for 96 samples run in a single lane using different multiplexing methods. Bars show standard error from the mean. Dashed boxes show simulated data for pre-capture 16 (see Results) in varying decreasing numbers of pools per lane. **A**) Coverage at 1X, 10X, and 20X, and **B**) Mean depth of coverage. * p < 0.01 (Independent samples *T*-test).

Depth of coverage represents the number of sequencing reads aligned over a sequenced base pair. On average, all methods of multiplexing showed a 1X coverage (Figure 3A) that was not significantly different: an average of 99.8%, 99.6%, and 99.4% for post-capture, pre-capture 12, and pre-capture 16, respectively. 10X depth of coverage (Figure 3A) was greater than 94% with all methods of multiplexing, but lowest for pre-capture 16 (97.4%, 96.9%, and 94.9% for post-capture, pre-capture 12, and pre-capture 16, respectively). The average depth of coverage for pre-capture 16 samples was significantly lower at 1X, 10X, and 20X than both post-capture and pre-capture 12 (p < 0.01 in all cases). Average depth of coverage (Figure 3B) was significantly reduced for pre-capture 12 (average = 196) and pre-capture 16 (average = 136) samples when compared with post-capture samples (average = 227) (p < 0.01 in all cases).

**Figure 4:**
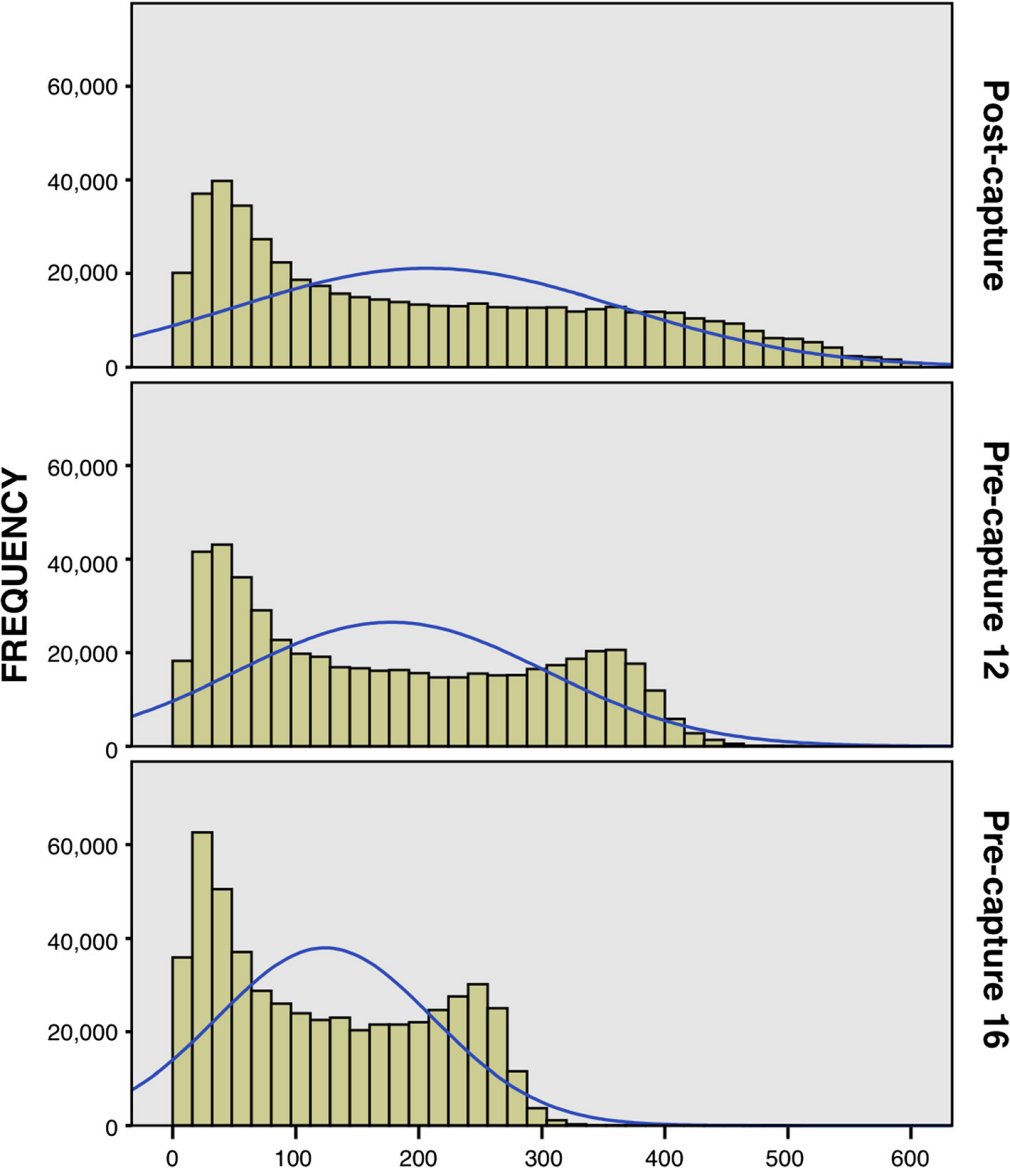
**Comparison of frequency histograms for average depth of coverage for each multiplexing method.** Histograms show the average depth of coverage results for 96 samples run in a single lane using different multiplexing methods with normal curve in blue.

We found a difference in coverage distribution among multiplexing methods (Figure 4). The distribution of coverage for post-capture multiplexing was most broadly distributed with increasingly sharply peaked distribution seen for pre-capture 12 and pre-capture 16 multiplexing. We identified no systematic differences in coverage of targeted regions that could not be accounted for by differences in total numbers of reads when comparing different methods of multiplexing.

In order to obtain a similar depth of coverage as post-capture samples when performing pre-capture 16 multiplexing, fewer pools of 16 can be sequenced in a single lane as a compensation for reduced capture efficiency. To test this hypothesis, we randomly sub-sampled sequencing reads from the pre-capture 16 lane to simulate a total of 5, 4, and 3 pools of 16 samples (80, 64, and 48 samples, respectively) per lane (Figure 3A and B). We validated this simulation technique by simulating data for 6 pools of 16 and found results were not significantly different when compared to actual data: average depth of coverage was 125X and 136X for simulated and actual data, respectively; coverage at 1X, 10X, and 20X was 99.5%, 96.5%, and 89.8% for simulated data and 99.7%, 94.9%, and 87.0% for actual data. Our simulations for fewer number of pools per lane showed that coverage levels approached post-capture averages when reduced by a single pool: coverage for 80 samples in 5 pools of 16 (80/5) was 99.7% at 1X, 97.6% at 10X and 92.6% at 20X (Figure 3). When the number of pools was further reduced, results surpassed post-capture averages.

### Variant detection

We used 96 unique DNA samples in three sets of 96 captures each. In each set of samples, 48 samples were from persons with presumed genetic hearing loss of an unknown cause and the results from these samples will be reported elsewhere. 48 samples in each set were either positive controls carrying deafness-causing mutations verified by Sanger sequencing or negative controls (Additional file 1: Table S1 and Additional file 2: Table S2). The exact composition of samples per set was different between post-capture and pre-capture sets due to sample limitations, but there was a similar composition of types of control mutations in each set. The post-capture positive control samples included 43 mutations: 5 small deletions, 2 large deletions, 27 missense mutations (34 heterozygous and 4 homozygous), 4 mitochondrial mutations, and 3 splice site mutations (all heterozygous). The two pre-capture multiplexing lanes contained the same 44 positive control mutations: 3 small deletions, 2 large deletions, 31 missense mutations (35 heterozygous and 2 homozygous), 6 mitochondrial mutations, and 3 splice site mutations (all heterozygous).

The average number of variants identified within the targeted regions of interest for post-capture, pre-capture 12, and pre-capture 16 samples was 601, 511, and 509 variants, respectively. When normalized by total number of sequencing reads per sample, there was no significant difference between methods (p = 0.642 and p = 0.677 for post-capture compared with pre-capture 12 and pre-capture 16, respectively). 100% of variants were identified in all three lanes using our variant calling and annotation pipeline (see Methods) with the exception of the sample from post-capture lane that failed at sequencing (Additional file 1: Table S1 and Additional file 2: Table S2). No pathogenic variants were identified in the negative controls. We examined allelic balance for heterozygous positive control variants and found no significant difference between post-capture, pre-capture 12, or pre-capture 16 samples (variant reads/total reads average [standard deviation] was 0.48 [0.02], 0.47 [0.02], 0.47 [0.02], respectively).

We performed a systematic analysis to examine the possibility of artifacts or sequencing errors associated with molecular barcoding that may lead to erroneous sample assignment (chimeras) and misdiagnosis. We searched within each lane for positive control variants present in the un-annotated (“raw”) variant call format (VCF) files for any of the samples present in any other sample in that lane even at low observation rates. We did not find any evidence of sample-switching due to aberrant barcode identification in any lanes, although we did find that a single individual was an incidental carrier for a disease-causing mutation in the *MYO7A* gene (p.Ala397Asp), as this variant was found in all three lanes and confirmed with Sanger sequencing.

### Advantages of pre-capture multiplexing

Pre-capture multiplexing was associated with reduced capture efficiency, which in turn reduced average depth of coverage but did not negatively impact variant detection. Significant savings in cost and time were associated with pre-capture multiplexing. Costs for XTv2 kits are 15% lower than XTv1. There are other costs that are associated with library preparation including consumables which are difficult to quantify, but can be estimated as 1/16^th^ or 1/12^th^ of the cost of post-capture TGE. As an example, each SPRI-bead purification during the protocol costs approximately $5.76/purification. Using pre-capture 16 multiplexing, samples are pooled for three of these purifications compared to post-capture TGE. For 96 samples, the cost simply for purification of each of these samples individually three times (288 purifications, as per the post-capture TGE method) is $1,658.88. Pre-capture 16 multiplexing would reduce the number of purifications required to 6 samples three times (18 purifications) and therefore the cost is $103.68 or a cost reduction of 93.75%. The same calculation yields a cost of $138.24 for pre-capture 12 multiplexing and a corresponding cost reduction of 91.7%. Similar reductions in cost can be assumed for other reagents and consumables.

In our hands, TGE requires 6 hours for pre-hybridization steps and 4 hours for post-hybridization steps. Post-capture multiplexing introduces barcodes at the final amplification step and pooling is completed immediately prior to sequencing, which does not reduce hands-on time. Pre-capture multiplexing occurs prior to the hybridization and therefore 12X or 16X as many samples can be hybridized and captured in the same amount of time. Therefore, though difficult to quantify, hands-on time is significantly reduced with pre-capture multiplexing.

## Discussion and conclusions

Massively parallel sequencing and TGE enable rapid and efficient sequencing of hundreds of thousands or millions of base pairs of the human genome simultaneously. While prices have decreased drastically, efficiency and cost-effectiveness are still important considerations, especially when large numbers of samples are analyzed. In this study we provide the first systematic comparison of post- and pre-capture multiplexing on a large sample set.

We used a large number of positive control mutations to validate this capture method, and in the process uncovered a variety of genomic variants including small and large deletions, and single nucleotide variants in control patients. We found no evidence of barcode switching or erroneous barcode assignment. In addition, the allelic balance, which is important for making heterozygous calls, was not affected by pre-capture pooling.

As expected, our data show that multiplexing samples prior to hybridization and capture reduces capture efficiency. Importantly, we noted a decrease in duplicate reads, which may partially offset this loss in efficiency. We used simulations to show that reduced efficiency can be compensated for by modifying the experimental design (i.e. reducing the total number of pools sequenced per lane). We hypothesize that this effect is due to competition for complementary RNA baits among multiple genomes, specifically because the effect was more pronounced with the higher pool size (pre-capture 16). In addition, we found a difference in coverage distribution when comparing multiplexing methods (Figure 4), with the distribution of coverage became less broad with pre-capture pooling. We believe this effect also reflects competition for baits with regions most deeply covered becoming distributed amongst multiple genomes when pre-capture multiplexing is used. Finally, we noted a significant reduction in total number of reads and percent of reads mapping. Because only 3 sequencing lanes were compared, it was not possible to determine whether this effect reflected inter-lane variability or the effects of pre-capture multiplexing.

Costs are an important factor in any experimental design. Kit costs for pre-capture multiplexing are 15% lower. However, greater cost savings are found in the reduction in consumables and ancillary reagents used when pre-capture multiplexing is employed. In our example, we show that costs of purifications alone are reduced by ~94% for pre-capture 16 multiplexing and ~92% for pre-capture 12 multiplexing. The most significant reduction when using pre-capture multiplexing lies in reduced hands-on time. Although this is difficult to quantify, 12X or 16X as many samples can be processed after pooling occurs when pre-capture multiplexing is used. Therefore, due to the greatest reduction in costs and hands-on time, as well as a lack of detrimental effects on quality of TGE, pre-capture 16 represents the most effective and efficient method for TGE of an experiment with a similar target size as shown here (521,647 bp). When designing their own experiments, investigators can estimate the optimum number of samples to pool pre-capture and sequence per lane based on the effects on capture efficiency described here.

Here we show for the first time the effect of pre-capture multiplexing during TGE on a large set of samples. We noted a specific effect on capture efficiency during pre-capture multiplexing and we hypothesize that this effect is due to multiple genomes competing for hybridization with complementary RNA. However, pre-capture multiplexing provided significant cost-savings and time reductions, resulted in no barcode mis-identification, and could reliably identify several classes of genetic variation. In summary, pre-capture multiplexing increases the efficiency of TGE and massively parallel sequencing to identify genomic variants.

## Methods

### Samples

This study was approved by the University of Iowa Institutional Review Board. We used 96 samples including DNA from the following individuals: 39 positive controls, 9 negative controls, and 48 unknowns. The positive control individuals had non-syndromic hearing loss (NSHL) and in total represented 44 mutations including small indels, large deletions and missense mutations previously diagnosed with Sanger sequencing (Additional file 1: Table S1 and Additional file 2: Table S2). We used 9 unaffected individuals or HapMap samples as negative controls. Due to sample quantities, the exact same set of control DNA could not be used twice. Instead, a similar composition of genetic variants was assembled to effectively compare post-capture and pre-capture multiplexing (Additional file 1: Table S1 and Additional file 2: Table S2). The individuals with unknown causes of hearing loss were enrolled in our large research study on deafness and results will be reported elsewhere.

### Targeted genomic enrichment and sequencing

We previously developed and reported our TGE platform, OtoSCOPE®, for diagnosis of genetic hearing loss [3]. The original design targeted 350,160 bp. We performed a systematic rebalancing of baits to improve on-target coverage, increase coverage over poorly covered areas (increase bait tiling), as well as decrease coverage variation among targets (reduce bait tiling over highly covered regions). The rebalanced baits targeted 521,647 bp. Eight base-pair error-correcting barcode sequences were adapted from another study [16], see Supplementary Information for barcode sequences, and no barcodes showed drop-out in our initial experiments (data not shown). We performed TGE here using liquid handling automation equipment (Bravo system, Agilent Technologies) using SureSelect XT version 1 (post-capture) or SureSelect XT version 2 (pre-capture 12 and pre-capture 16) kits. All sequencing was performed on the Illumina HiSeq 2000 using paired-end 76 bp reads with 96 samples sequenced per lane.

### Bioinformatics analysis

Bioinformatics analysis was performed using a local installation of the Galaxy software and a custom analysis pipeline, as previously described [3] with modifications. Briefly, read mapping to the human reference genome (Hg19) was performed using BWA [17]; duplicate reads were analyzed and removed with Picard (http://picard.sourceforge.net); and local realignment and variant identification was completed with GATK [18]. We used a threshold of 4 sequencing reads and required a variant to be present in ≥15% of reads to be considered. Variants were compared against our database of deafness variants (deafnessvariationdatabase.org). Statistics were calculated using Samtools [19], Bedtools [20] and NGSRich [21]. Copy number variants (CNVs) were determined using a previously published method that normalizes sequencing depth among samples, identifies outliers, and calls via a sliding-window method [22].

## Abbreviations

TGE: Targeted genomic enrichment; MPS: Massively parallel sequencing.

## Competing interests

SJ, HR, ACG, BN, SH, and EML are employees of Agilent Technologies Inc., which manufactures and offers for commercial sale targeted genomic enrichment kits like the one used in this work. AES, MSH, and RJHS have no competing financial interests.

## Authors’ contributions

AES, MSH, RJHS, SH, and EML designed the experiment. AES, MSH, HR, and SJ performed the experiments. BN designed the sequence capture baits. AES, SJ, HR, ACG, and RJHS analyzed the data. AES, MSH, and RJHS drafted the manuscript. All authors read and approved the final manuscript.

## Supplementary Material

Additional file 1 **Table S1.** Positive Control Mutations. Click here for file

Additional file 2 **Table S2.** 8 bp barcodes used. Click here for file
