# Supplementary material for: Pre-capture multiplexing improves efficiency and cost-effectiveness of targeted genomic enrichment
Source: BMC Genomics. 2012 Nov 14;13:618. doi: 10.1186/1471-2164-13-618 (PMC3534602; doi:10.1186/1471-2164-13-618)
Supplement: Additional file 2 — Table S2. 8 bp barcodes used. [file 1471-2164-13-618-S2.pdf]

Index #	INDEX SEQ
#1	AACGTGAT
#2	AAACATCG
#3	ATGCCTAA
#4	AGTGGTCA
#5	ACCACTGT
#6	ACATTGGC
#7	CAGATCTG
#8	CATCAAGT
#9	CGCTGATC
#10	ACAAGCTA
#11	CTGTAGCC
#12	AGTACAAG
#13	AACAACCA
#14	AACCGAGA
#15	AACGCTTA
#16	AAGACGGA
#17	AAGGTACA
#18	ACACAGAA
#19	ACAGCAGA
#20	ACCTCCAA
#21	ACGCTCGA
#22	ACGTATCA
#23	ACTATGCA
#24	AGAGTCAA
#25	AGATCGCA
#26	AGCAGGAA
#27	AGTCACTA
#28	ATCCTGTA
#29	ATTGAGGA
#30	CAACCACA
#31	CAAGACTA
#32	CAATGGAA
#33	CACTTCGA
#34	CAGCGTTA
#35	CATACCAA
#36	CCAGTTCA
#37	CCGAAGTA
#38	CCGTGAGA
#39	CCTCCTGA
#40	CGAACTTA
#41	CGACTGGA
#42	CGCATACA
#43	CTCAATGA
#44	CTGAGCCA
#45	CTGGCATA
#46	GAATCTGA

#47	GACTAGTA
#48	GAGCTGAA
#49	GATAGACA
#50	GCCACATA
#51	GCGAGTAA
#52	GCTAACGA
#53	GCTCGGTA
#54	GGAGAACA
#55	GGTGCGAA
#56	GTACGCAA
#57	GTCGTAGA
#58	GTCTGTCA
#59	GTGTTCTA
#60	TAGGATGA
#61	TATCAGCA
#62	TCCGTCTA
#63	TCTTCACA
#64	TGAAGAGA
#65	TGGAACAA
#66	TGGCTTCA
#67	TGGTGGTA
#68	TTCACGCA
#69	AACTCACC
#70	AAGAGATC
#71	AAGGACAC
#72	AATCCGTC
#73	AATGTTGC
#74	ACACGACC
#75	ACAGATTC
#76	AGATGTAC
#77	AGCACCTC
#78	AGCCATGC
#79	AGGCTAAC
#80	ATAGCGAC
#81	ATCATTCC
#82	ATTGGCTC
#83	CAAGGAGC
#84	CACCTTAC
#85	CCATCCTC
#86	CCGACAAC
#87	CCTAATCC
#88	CCTCTATC
#89	CGACACAC
#90	CGGATTGC
#91	CTAAGGTC
#92	GAACAGGC
#93	GACAGTGC

#94	GAGTTAGC
#95	GATGAATC
#96	GCCAAGAC
